# Left Purkinje premature ventricular complexes following left bundle branch area pacing

**DOI:** 10.1016/j.hrcr.2022.03.017

**Published:** 2022-03-25

**Authors:** Thibault Lenormand, Arnaud Bisson, Alexandre Bodin, Dominique Babuty, Nicolas Clementy

**Affiliations:** ∗ Department of Cardiology, University Hospital of Tours, Tours, France; † Department of Cardiology, Hospital Centre of Orléans, Orléans, France

**Keywords:** Left bundle branch area pacing, Premature ventricular complex, Purkinje fibers, Atrioventricular node ablation, Resynchronization

**Key Teaching Points**

• Left bundle branch area pacing is a recent technique using a ventricular transseptal approach to try and capture the left bundle branch and achieve physiological pacing and electrical synchrony.
• The presence of spontaneous ventricular complexes in patients having undergone atrioventricular node ablation must be explored to determine whether there is a repermeabilization of the atrioventricular node or spontaneous premature ventricular complexes (PVCs).
• PVCs with right bundle branch block morphology and a rapid initial depolarization are suggestive of Purkinje-related complexes.
• Patients undergoing left bundle branch area pacing and atrioventricular node ablation may be exposed to Purkinje-related PVCs.

## Introduction

Left bundle branch area pacing (LBBAP) is defined as a capture of the left bundle branch, usually associated with deep septal myocardium capture, using a ventricular transseptal approach.

LBBAP is a promising technique for delivering physiological pacing to achieve electrical synchrony, and may become an option for cardiac resynchronization in patients with heart failure and a reduced left ventricular ejection fraction presenting with left bundle branch block. However, data on chronic complications are still lacking.

Here we report the cases of 3 patients with procedure-related premature ventricular complexes (PVCs) following LBBAP.

## Case report

### Case 1

A 67-year-old man with a history of symptomatic persistent atrial fibrillation despite pulmonary vein isolation and optimal antiarrhythmic therapy, complicated with a reduced left ventricular ejection fraction of 40%, and normal coronary arteries, underwent the implantation of a single-chamber pacemaker with LBBAP, followed by atrioventricular node ablation.

Left bundle branch pacing was performed using a SelectSecure 3830 lead (Medtronic, Minneapolis, MN). Radiofrequency atrioventricular node ablation was then performed, resulting in a complete atrioventricular block. The pacemaker was then set to a VVIR mode with a rest frequency of 75 beats per minute.

**Figure 1:**
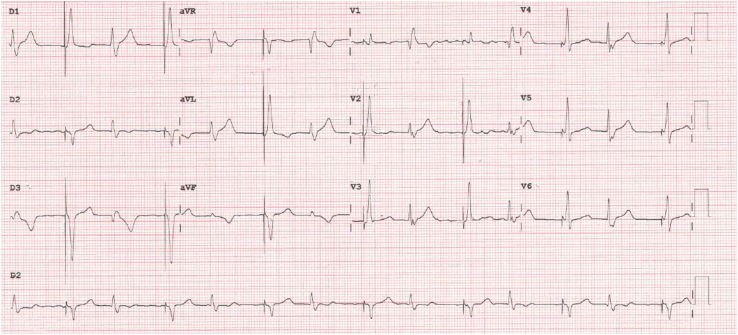
Patient 1, 12-lead electrocardiogram. Left bundle branch area pacing with left axis alternates with ventricular bigeminy with a right bundle branch block morphology, suggesting an origin in the left Purkinje network.

The patient presented 1 month later at the emergency department for palpitations. Physical examination was normal except for irregular pulse. The patient’s 12-lead electrocardiogram (Figure 1) showed atrial fibrillation with ventricular pacing consistent with LBBAP, and spontaneous monomorphic ventricular bigeminy with a right bundle branch pattern and a rapid initial depolarization suggestive of Purkinje-related complexes.

Pacemaker interrogation showed a persistent complete atrioventricular block, confirming the diagnosis of PVCs and absence of repermeabilization of the atrioventricular node. Pacemaker interrogation showed a 20% burden of sensed ventricular complexes.

### Case 2

An 80-year-old woman with a history of symptomatic persistent atrial fibrillation and atrial tachycardia despite antiarrhythmic therapy, electrical cardioversion, and 2 ablative procedures underwent the implantation of a single-chamber pacemaker followed by atrioventricular node ablation as a means to control heart rate. Considering the risk of left ventricular dyssynchrony consecutive to the pacemaker implantation with permanent right ventricular pacing, it was decided to perform LBBAP using a SelectSecure 3830 lead.

Radiofrequency atrioventricular node ablation was then performed, resulting in a complete atrioventricular block, with subsequent setting of the pacemaker to a VVIR mode with a resting frequency of 75 beats per minute.

The patient presented to routine follow-up consultation 6 months later, complaining of recurring palpitations, without dyspnea, chest pain, or any other symptom. Physical examination was normal except for an irregular pulse.

**Figure 2:**
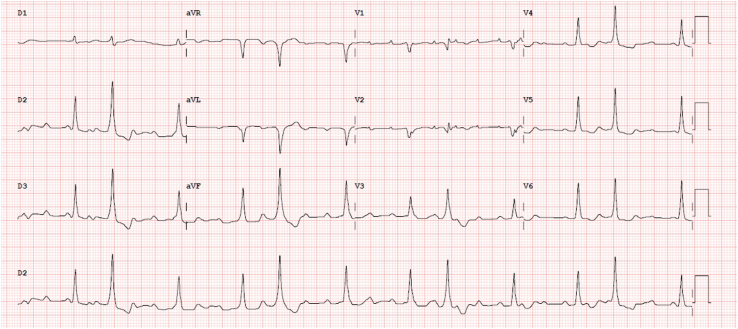
Patient 2, 12-lead electrocardiogram. Left bundle branch area pacing with vertical axis alternates with ventricular trigeminy with a similar right bundle branch block pattern, suggesting an origin in the left anterior Purkinje network.

The patient’s 12-lead electrocardiogram (Figure 2) showed an atrial tachycardia with ventricular pacing consistent with LBBAP, spontaneous monomorphic ventricular trigeminy with a right bundle branch block morphology, and a rapid initial depolarization typical of Purkinje-related complexes.

Pacemaker interrogation showed a persistent complete atrioventricular block, confirming the diagnosis of PVCs and absence of repermeabilization of the atrioventricular node, and a 13% burden of sensed ventricular complexes.

### Case 3

An 80-year-old woman with a history of symptomatic persistent atrial fibrillation despite electrical cardioversion, surgical treatment of atrial fibrillation during a coronary bypass surgery, and optimal antiarrhythmic therapy underwent the implantation of a pacemaker followed by atrioventricular node ablation to control heart rate. Considering the risk of cardiac dyssynchrony consecutive to the pacemaker implantation with permanent stimulation, it was decided to perform LBBAP using a SelectSecure 3830 lead.

Radiofrequency atrioventricular node ablation was then performed, resulting in a complete atrioventricular block, with subsequent setting of the pacemaker to a VVIR mode with a resting frequency of 75 beats per minute.

Intermittent failure to capture occurred during immediate follow-up, leading to subsequent changing of the LBBAP lead after 2 days.

The patient presented to routine follow-up 6 months later, without any cardiovascular symptoms. Physical examination was normal except for an irregular pulse.

**Figure 3:**
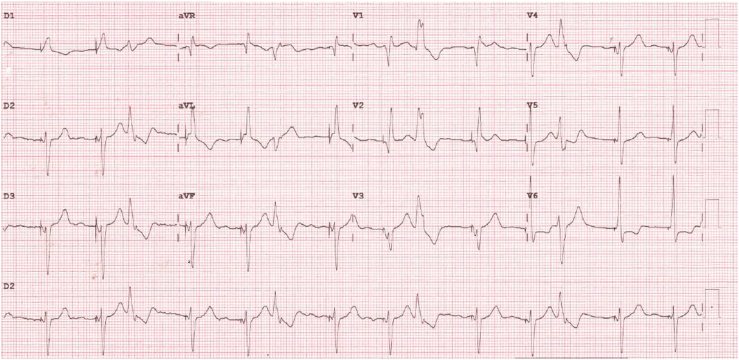
Patient 3, 12-lead electrocardiogram. Left bundle branch area pacing with left axis alternates with ventricular trigeminy with a right bundle branch block pattern and vertical axis, suggesting an origin in the left anterior Purkinje network.

The patient’s 12-lead electrocardiogram (Figure 3) showed atrial fibrillation with ventricular pacing consistent with LBBAP, spontaneous monomorphic ventricular trigeminy with a right bundle branch block morphology, and a rapid initial depolarization suggestive of Purkinje-related complexes.

Pacemaker interrogation showed a persistent complete atrioventricular block, confirming the diagnosis of PVCs and absence of repermeabilization of the atrioventricular node.

## Discussion

Given that it is a recent approach for cardiac stimulation and resynchronization therapy, long-term effects and complications of LBBAP are still being discovered, and these 3 similar cases highlight a previously unknown phenomenon upon these patients’ follow-up.

We hypothesize that left Purkinje-related PVCs occurring in left bundle branch pacing could be related to a direct reentrant mechanism, as it has previously been described with bundle branch reentrant ventricular tachycardia during LBBAP.^1^ Though the precise underlying mechanism remains unclear, it seems plausible that the apparition of left Purkinje PVCs consecutive to left bundle branch pacing could be the consequence of a direct reentrant mechanism caused by the left bundle branch stimulation in the same manner.

Another possible explanation may be the creation of local Purkinje injury during lead implantation and repositioning, constituting the substratum for PVCs. Left bundle branch pacing uses a transseptal approach, where the lead traverses the septum before reaching the left bundle branch area, which could cause local trauma to the Purkinje fibers.

Guidance of left bundle branch pacing by PVCs has been well described,^2^ with PVC morphology alterations as the lead penetrates the septum. In this case series, PVCs with a right bundle branch block morphology and relatively narrow QRS complexes predicted the left bundle capture. This morphology is consistent with the ones observed in our 3 patients.

Another hypothesis could be that rate control achieved by atrioventricular node ablation could allow the presentation of PVCs that were so far suppressed by the rapid ventricular rate consecutive to atrial fibrillation.

Of course, one cannot exclude a role of atrioventricular node ablation on occurrence of Purkinje PVCs; however, no data were found in the literature suggesting any link. Ablation was performed proximal to the His bundle, in the fast AV node pathway area, and premature complexes’ origins are distal to the bifurcation of the His bundle, specifically on the left bundle branch, which was targeted for pacing.

These 3 cases highlight a potential risk of LBBAP-induced left-Purkinje PVCs. Although the underlying mechanism remains unclear, the similarities between the 3 patients and the consistent morphology with previous descriptions in other case reports stress the need for careful follow-up of LBBAP patients, both for screening for these PVCs and for assessing their long-term impact, especially on left ventricular systolic function. Therapeutic strategy and potential proarrhythmic risk remain unknown.

In our experience of more than 100 LBBAP implantations, one-third of them having undergone atrioventricular node ablation, only 3 such cases occurred. Further descriptions and data are required to determine the prevalence of PVC following left bundle branch pacing, and cardiac imaging and electrophysiological studies may be helpful to precise the underlying mechanism.

## Conclusion

Patients with left bundle branch pacing and atrioventricular ablation may be exposed to left Purkinje-related PVCs. The underlying mechanism, prevalence, and long-term consequences need to be explored.
